# Fees from a Pasha

**Published:** 1846-12

**Authors:** 


*Fees from a Pasha.*—If it be true that next to the gratification desired from the conciousness of wealth, is the pleasure of contemplating the riches of our neighbors, (?) then we republican physicians may take comfort in the advantages which our European brethren possess in having nobles, princes and crowned heads for patients! *Ed. Buffalo Jour.*

"According to the *Gazette Medicale*, M. Lallemand did not fix any sum for medical attendance on Ibrahim Pasha, during his residence in the south of France. Ibrahim himself proposed paying down 100,000 francs, (£4000;) and early in May he sent his secretary with this sum, for which M. Lallemand gave a receipt, without making any remark, but obviously with a discontented air. When Ibrahim was informed of this he sent another 50,000 francs, making in the whole 150,000 francs, or £6000! It is reported that a smile of satisfaction now began to appear upon the face of this fortunate son of Esculapius; but this grasping at fees appears to have led to a rupture between Ibrahim and a relative of M. Lallemand's, who was about to receive a lucrative appointment from the Prince, and to accompany him to Egypt.

Out of Scylla into Charybdis. So soon as Ibrahim had freed himself from the extortion of a surgeon he fell into the hands of a Parisian dentist, whom he had unfortunately employed to make four teeth for him. Ibrahim thought that a payment of 6000 francs, (£240) would be a sufficient renumeration. M. Rogers, the dentist, whose name has already figured in these pages, probably thinking that he might not again have an Egyptian prince for a patient, took this sum only as an instalment, and gave a receipt, which the secretary mistook for a complete acquittance. In about a week afterwards the enterprising dentist sent in a charge of 9000 francs, (£360) for a set of teeth! In the impulse of the moment Ibrahim said that to spend so much money on his mouth was literally eating up his fortune, and he declined paying. He was threatened with law proceedings, when he compromised the matter by paying 3000 francs, (£120.)

As these statements appear in a respectable medical journal, we suppose we must take them as true. If so, Ibrahim is not likely to forget his French surgeon and dentist, who may very appositely be described as first-rate *ecorcheurs*."—*Lon. Med. Gazette.*

